# Low-Complexity System and Algorithm for an Emergency Ventilator Sensor and Alarm

**DOI:** 10.1109/TBCAS.2020.3020702

**Published:** 2020-09-01

**Authors:** Ryan M. Corey, Evan M. Widloski, David Null, Brian Ricconi, Mark A. Johnson, Karen C. White, Jennifer R. Amos, Alexander Pagano, Michael L. Oelze, Rachel D. Switzky, Matthew B. Wheeler, Eliot B. Bethke, Clifford F. Shipley, Andrew C. Singer

**Affiliations:** 1 University of Illinois at Urbana-Champaign 14589 Urbana IL 61801 USA; 2 Creative Thermal Solutions, Inc. Urbana IL 61802 USA; 3 Carle Health Urbana IL 61801 USA

**Keywords:** Biomedical monitoring, biomedical signal processing, envelope detectors, pressure measurement, ventilators

## Abstract

In response to anticipated shortages of ventilators caused by the COVID-19 pandemic, many organizations have designed low-cost emergency ventilators. Many of these devices are pressure-cycled pneumatic ventilators, which are easy to produce but often do not include the sensing or alarm features found on commercial ventilators. This work reports a low-cost, easy-to-produce electronic sensor and alarm system for pressure-cycled ventilators that estimates clinically useful metrics such as pressure and respiratory rate and sounds an alarm when the ventilator malfunctions. A low-complexity signal processing algorithm uses a pair of nonlinear recursive envelope trackers to monitor the signal from an electronic pressure sensor connected to the patient airway. The algorithm, inspired by those used in hearing aids, requires little memory and performs only a few calculations on each sample so that it can run on nearly any microcontroller.

## I. Introduction

The COVID-19 crisis may cause shortages of ventilators used to treat patients with severe respiratory symptoms [1]. COVID-19 patients can experience acute respiratory distress syndrome (ARDS), which causes extreme difficulty breathing due to fluid leaking into the lungs [2]–[4]. Mechanical ventilation can help to treat these patients by providing oxygen while the underlying disease runs its course [2], [5], [6]. Appropriate oxygen delivery is a mainstay of critical care and in COVID-19 can prevent death from ARDS and hypoxemia.

Because the growing number of COVID-19 cases may exceed the number of available ventilators, dozens of companies, university research teams, and other organizations have developed emergency ventilators under special authorizations from regulators [7], [8]. Pressure-cycled pneumatic ventilators, like the Illinois RapidVent developed by the authors’ institutions [9], [10], are especially attractive for this emergency because they can be rapidly and inexpensively manufactured. They are powered by pressurized gas and controlled by a mechanical modulator, so they require no electronic components to operate [11]–[13]. However, they lack the sensors found in more-expensive commercial ventilators that provide closed-loop control, monitoring, and alarm capabilities. Clinicians rely on these electronic systems to adjust ventilator settings and to alert them to ventilator malfunctions or patient activity that require their attention. Without sensing and alarm features, clinicians must constantly monitor each patient and cannot be sure that ventilator settings are correct.

This work describes an electronic sensor and alarm system for pressure-cycled emergency ventilators. Like the pneumatic ventilators it is designed to complement, this device must be of low cost and must be easy to produce from readily available components. The most important function of the device is to sound an alarm when the breathing cycle is abnormal. Because pressure-cycled ventilators use pressure levels to switch between inhalation and exhalation modes, they produce distinctive pressure waveforms [14], [15]. The sensor and alarm system can analyze this pressure signal to determine whether the ventilator is cycling normally. The same pressure signal can be used to detect sudden pressure loss due to disconnection and pressure spikes due to mechanical failure and to estimate clinically useful parameters such as the peak inspiratory pressure (PIP), positive end-expiratory pressure (PEEP), and respiratory rate (RR).

These sensing and alarm functions reproduce most of the functionality of similar commercial monitoring products. A full-featured monitoring system would also measure tidal volume, or the amount of air delivered with each breath, and the oxygen concentration in the air, and it would trigger alarms based on these metrics [6], [8], [13]. However, tidal volume and oxygen concentration cannot be inferred from the pressure waveform alone and would require more complex equipment.

**Fig. 1.**
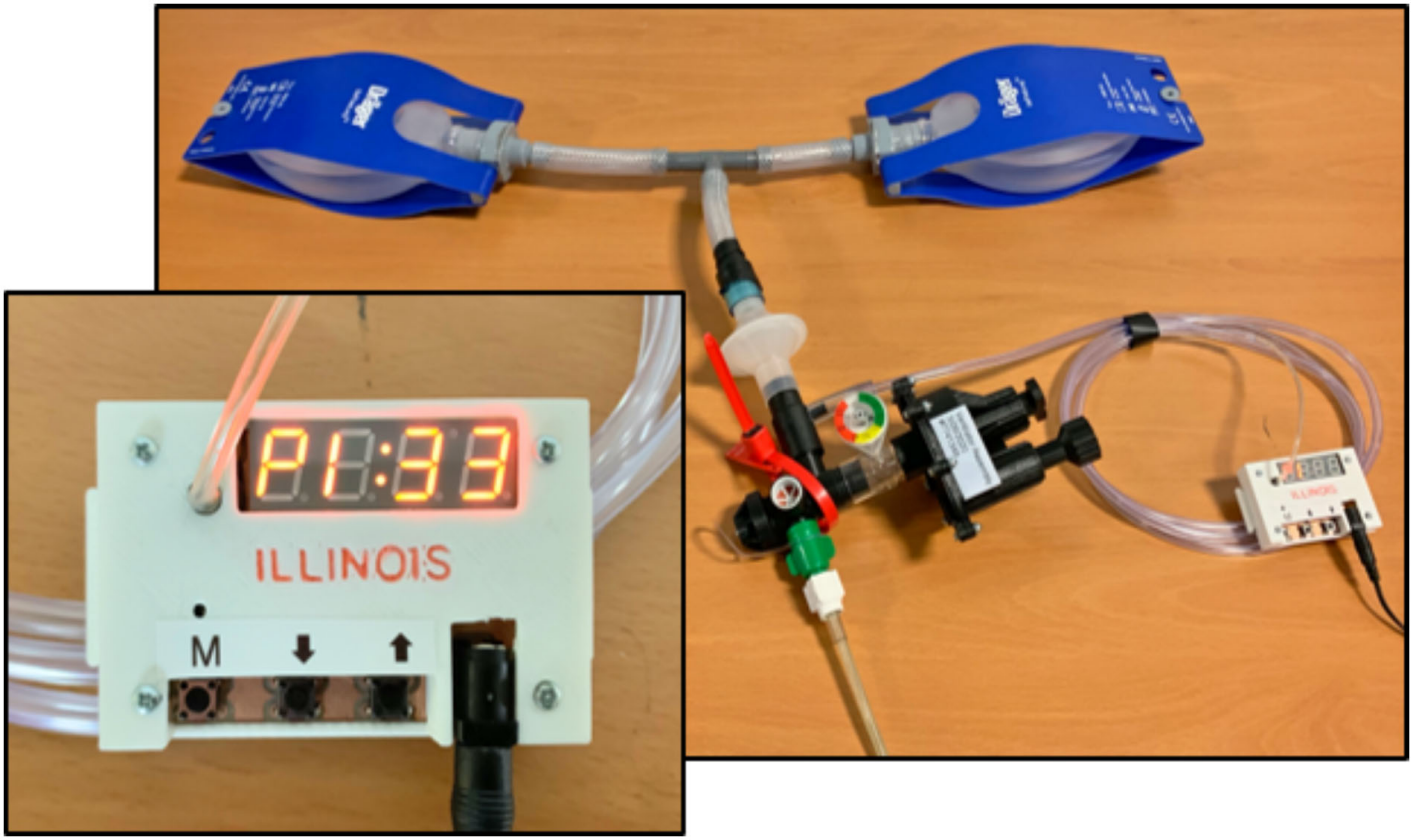
Prototype of the Illinois RapidAlarm sensor and alarm system attached to a pressure-cycled ventilator and artificial lung.

The device reported here, known as the Illinois RapidAlarm and shown in Fig. 1, monitors the pressure-cycled ventilator using an electronic pressure sensor that connects to the patient airway using a standard respiratory tubing adapter. A microcontroller analyzes the pressure signal using low-complexity signal processing algorithms inspired by audio processing methods used in hearing aids. Because the proposed algorithm does not store past samples of the signal in memory and performs only a few calculations on each sample, it can run on nearly any microcontroller. The hardware design files and software code for the Illinois RapidAlarm are available online under open-source licenses.^1^^1^[Online]. Available: https://rapidalarm.github.io. This work describes the design of the system, with particular attention to the signal processing algorithm used to estimate breathing metrics and detect malfunctions. The algorithm is validated using animal data and a hardware prototype is demonstrated using an artificial lung.

## II. Pressure-Cycled Ventilation

**Fig. 2.**
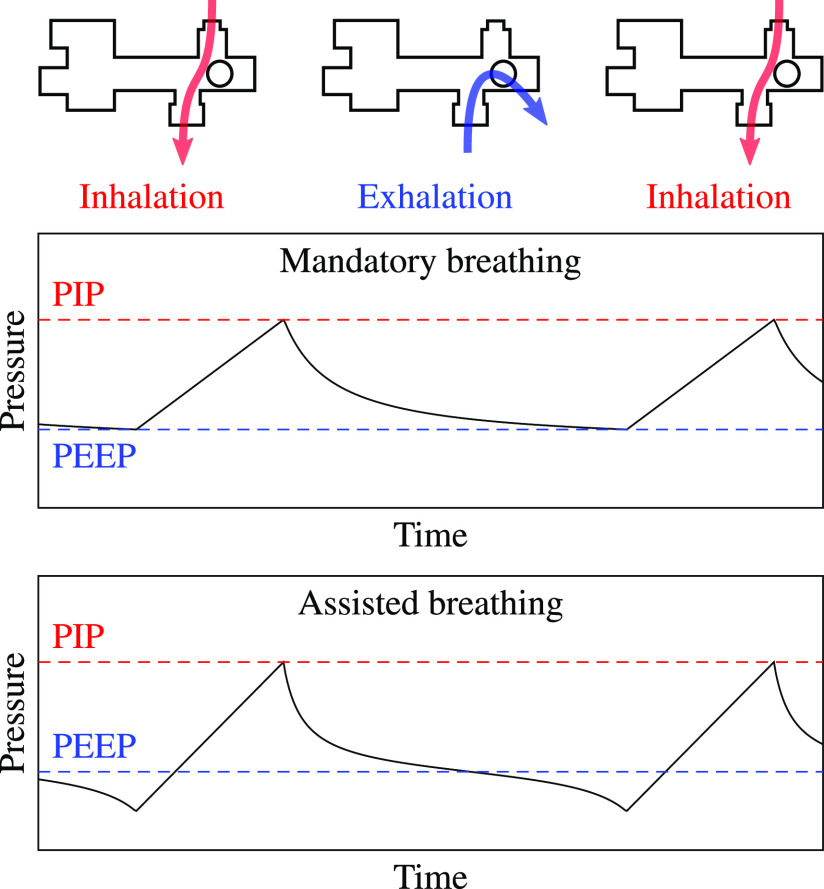
A pressure-cycled ventilator uses positive pressure to deliver gas to the patient airway. During normal operation, it produces a distinctive pressure waveform.

Pressure-cycled pneumatic ventilators, which are powered by pressurized gas, are useful in the present emergency because they have low cost, are easy to manufacture, and require no electronic components for basic operation. They provide pressurized gas to the patient airway and cycle between inhalation and exhalation modes using a pressure-switching mechanism controlled by pneumatic logic, as shown in Fig. 2. During inhalation, high-pressure gas flows from the ventilator to the patient's lungs. As the lungs inflate, the pressure in the airway increases until it reaches the peak inspiratory pressure (PIP), a maximum pressure threshold that can be adjusted by the user.

Once PIP is reached, the modulator opens a path to the atmosphere that allows air from the lungs to exit the ventilator. During exhalation, the pressure in the airway drops steadily, but does not fall to atmospheric pressure. Instead, once it drops below the positive end-expiratory pressure (PEEP), a spring closes the path to the atmosphere to initiate the next inhalation. During assisted-breathing mode, also known as pressure-support mode, the patient initiates a breath by inhaling to pull the pressure below the PEEP threshold. The clinician can control flow by adjusting a PIP dial and a rate dial, which determines expiratory time.

In pneumatic ventilators, the PEEP threshold is a fixed fraction of PIP determined by the mechanical design of the device. Because COVID-19 patients can require PEEP levels in the range 10--15 cm H$_{2}$O and PIP levels in the range 30--40 cm H$_{2}$O [4], some COVID-19 emergency ventilators are designed with smaller PIP-to-PEEP ratios than commercial ventilators. For example, the Illinois RapidVent has a measured PIP-to-PEEP ratio of about 2.4.

Pressure-cycled ventilators produce characteristic pressure waveforms, as shown in Fig. 2. During mandatory breathing, the airway pressure rises from PEEP to PIP during inspiration, then drops from PIP to PEEP during exhalation. During assisted breathing, the basic shape is the same, but the pressure may fall below PEEP when the patient initiates inhalation. The pressure signal can be used to estimate the PIP, PEEP (or minimum pressure for assisted breathing), and RR.

Because pressure-cycled ventilators produce well-defined pressure waveforms during normal operation, the pressure signal can also be used to detect malfunctions. If the gas circuit becomes obstructed or disconnected, the modulator will stop cycling between inhalation and exhalation modes, causing the pressure signal to remain constant. The proposed alarm system uses low-complexity signal processing algorithms to detect this constant-pressure condition.

## III. Sensing System

To ensure that it is broadly useful in as many settings as possible, the sensor and alarm system is not designed to be integrated with any particular model of ventilator. Rather, it is a standalone component that can be attached to any pressure-cycled ventilator. Because it is intended to address an emergency shortage, the design prioritizes cost and ease of production. The system uses low-cost, widely available parts, can be assembled on a two-layer printed circuit board using either through-hole or surface-mount components, and runs on a standard 5 volt power supply.

**Fig. 3.**
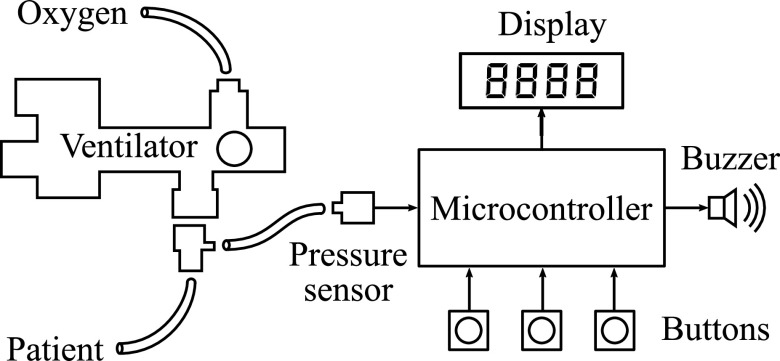
An electronic pressure sensor monitors gas pressure in the patient airway. A low-power microcontroller processes the pressure signal to generate measurements and alarms.

The sensor and alarm system is shown in Fig. 3. The device connects to the patient airway using standard respiratory tubing adapters attached on the patient side of the ventilator. The electronic system consists of a microcontroller, a display module, push buttons, a buzzer, and a pressure sensor. Our implementation uses the 8-bit microcontroller ATmega328, which was selected for its ease of use and wide availability. It is driven by an internal 8 MHz clock and does not require an external oscillator. Because the computational requirements of the proposed algorithm are low, as explained in Section VII, nearly any microcontroller with an analog-to-digital converter and several digital inputs and outputs should be suitable for the sensor and alarm module. The open-source firmware provides a hardware-agnostic C implementation of the monitoring algorithm that can be ported to other systems.

The user interface consists of three buttons and a four-character seven-segment display. In display mode, the display cycles through the three metrics (PIP, PEEP, and RR) every few seconds. The buttons are used to enable and disable the alarm and to adjust user-configurable alarm settings, which are described in Section VI. The alarm itself is a 4 kHz piezoelectric buzzer.

**TABLE I:** Alarm Conditions

Alarm	Condition	Tunable range on RapidAlarm
High pressure	$p[t]>p_{\mathrm{max}}$	$30\leq p_{\mathrm{max}}\leq 90$ cm H$_{2}$O
Low pressure	$p[t]< p_{\mathrm{min}}$	$1\leq p_{\mathrm{min}}\leq 20$ cm H$_{2}$O
High RR	$\mathrm{RR}[n]>\mathrm{RR}_{\mathrm{max}}$	$15\leq \mathrm{RR}_{\mathrm{max}}\leq 60$ breath/min
Low RR	$\mathrm{RR}[n]< \mathrm{RR}_{\mathrm{min}}$	$5\leq \mathrm{RR}_{\mathrm{min}}\leq 15$ breath/min
Noncycling	\begin{align*} t-T_{\mathrm{high}}[n] & >T_{\mathrm{max}}\\ t-T_{\mathrm{low}}[n] & >T_{\mathrm{max}}\\ v_{\mathrm{high}}[t]/v_{\mathrm{low}}[t] & < r_{\mathrm{min}}\\ v_{\mathrm{high}}[t]\!-\!v_{\mathrm{low}}[t] & < d_{\mathrm{min}} \end{align*}	$\,\,5\leq \frac{T_{\mathrm{max}}}{f_s} \leq 30$ sec

The key component in the system is the pressure sensor. The sensor interfaces with the patient airway via a tube and converts pressure levels into electrical signals, which are transmitted to the analog-to-digital converter on the microcontroller. To capture the range of pressure levels produced by pressure-cycled ventilators, the sensor should have a range of at least 0 to 50 cm H$_{2}$O. In our implementation, we used the NXP MPXV5010 piezoresistive pressure sensor, which has a pressure range of about 0 to 100 cm H$_{2}$O and provides output voltages from about 0 to 5 volts.

## IV. Pressure Tracking

The behavior of pressure-cycled ventilators is well characterized by the pressure signal measured at the patient airway. During normal operation, the pressure cycles between PIP and PEEP once per breath, as shown in Fig. 2. In an ideal system, breaths could be tracked by simply finding maxima and minima in this signal. However, real signals do not always increase and decrease monotonically like the waveform in that figure. The tracking algorithm must be robust against small pressure variations and must have low computational requirements so that it can run on inexpensive, low-power microcontrollers.

**Fig. 4.**
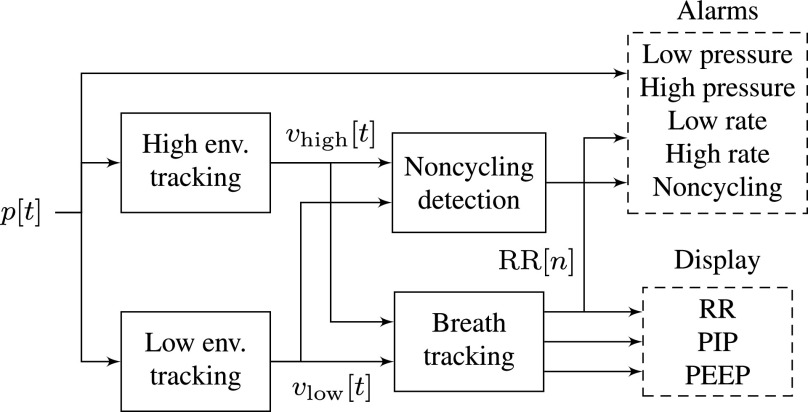
Alarm conditions and clinical metrics are derived from the measured pressure signal.

**Fig. 5.**
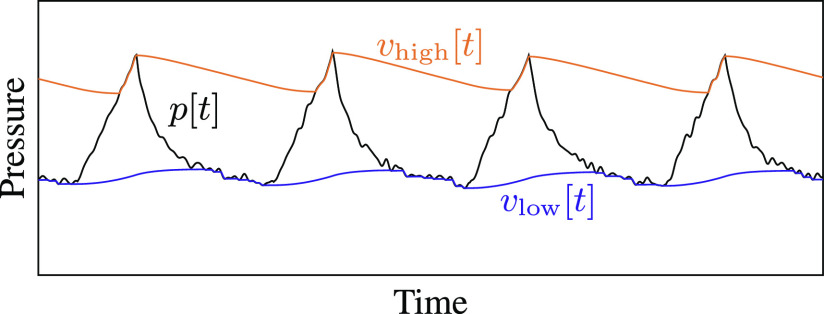
A pair of recursive peak detectors track the envelope of the pressure signal without storing past samples in memory.

The proposed processing system, illustrated in Fig. 4, uses a pair of nonlinear recursive filters to track the envelope of the pressure signal. Recursive filters of the form $y[t]=ay[t-1]+bx[t]$ are widely used in signal processing for their computational efficiency: because they use feedback from the output to the input of the filter, they can perform many filtering tasks with less memory and fewer multiplications than feed-forward filters [16]. Envelope tracking uses a nonlinear version of this recursive filter: each performs a moving average, but gives more weight to changes in one direction than another. The high-pressure envelope increases quickly but decreases slowly, so it follows the top of the pressure signal, while the low-pressure envelope decreases quickly and increases slowly, following the bottom of the signal, as illustrated in Fig. 5. Envelope detection is widely used as part of dynamic range compression in music production and in digital hearing aids, which also have severe computational constraints [17], [18].

Let $p[t]$ be the discrete-time pressure signal from the sensor, where $t$ is the sample index. The high-pressure envelope $v_{\mathrm{high}}[t]$ and the low-pressure envelope $v_{\mathrm{low}}[t]$ are given by

\begin{align*}
v_{\mathrm{high}}[t] & \!=\!{\begin{cases}\alpha _{A}v_{\mathrm{high}}[t\!-\!1]+(1\!-\!\alpha _{A})p[t], & \!\text{if }p[t]\geq v_{\mathrm{high}}[t\!-\!1]\\
\alpha _{R}v_{\mathrm{high}}[t\!-\!1]+(1\!-\!\alpha _{R})p[t], & \!\text{if }p[t]< v_{\mathrm{high}}[t\!-\!1] \end{cases}} \tag{1}
\\
v_{\mathrm{low}}[t] & \!=\!{\begin{cases}\alpha _{A}v_{\mathrm{low}}[t\!-\!1]+(1\!-\!\alpha _{A})p[t], & \!\text{if }p[t]\leq v_{\mathrm{low}}[t\!-\!1]\\
\alpha _{R}v_{\mathrm{low}}[t\!-\!1]+(1\!-\!\alpha _{R})p[t], & \!\text{if }p[t]>v_{\mathrm{low}}[t\!-\!1], \end{cases}} \tag{2}
\end{align*}

where $\alpha _{A}\in [0,1]$ and $\alpha _{R}\in [0,1]$ are called the attack coefficient and release coefficient, respectively. These coefficients determine the relative importance of old and new samples in the moving average; they control how quickly the envelope tracker responds to changes in the pressure signal.

When the tracker is responding rapidly to a change in signal level (an increase for the high-pressure envelope or a decrease for the low-pressure envelope), it is said to be in attack mode. The attack coefficient $\alpha _{A}$ is relatively small so that the tracker quickly forgets past estimates and follows the new sample. For example, in our implementation at 100 samples/sec, $\alpha _A = 0.9$ so that the $1/e$ decay time of past samples in attack mode is around 100 ms.

When the tracker is responding slowly (to a decrease in pressure for the high-pressure envelope or an increase for the low-pressure envelope), it is said to be in release mode. The release coefficient $\alpha _{R}$ is closer to 1 so that the envelope decays more slowly, allowing the algorithm to ignore small fluctuations in pressure within a single breath cycle. The rate of decay in release mode should be slow enough that the high and low envelopes stay far apart during a normal breath cycle. If the tracker is too slow, however, it could miss breaths when the pressure settings are adjusted or, worse, might take too long to trigger an alarm when the ventilator stops working. The choice of $\alpha _{R}$ is discussed further in Section VI-C.

Notice that each recursive envelope tracker need only store one previous envelope value in memory. For comparison, a system using a rolling maximum/minimum filter approach at a sample rate of 100 samples per second would need to store about 200 past measurements for a window size of two seconds.

## V. Ventilation Monitoring

**Fig. 6.**
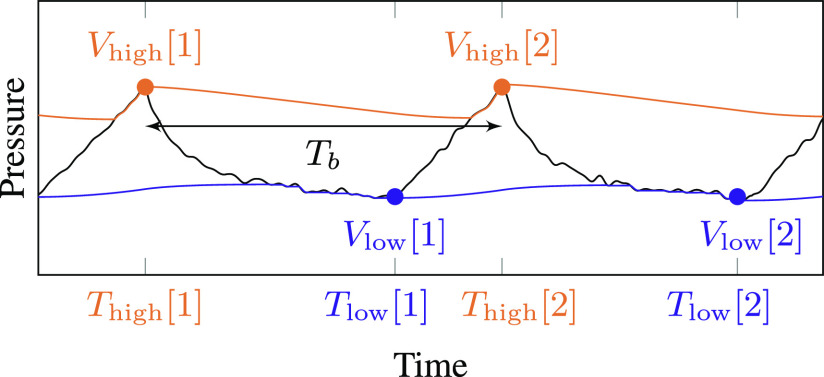
The inhalation/exhalation state of the ventilator is inferred from attack events on the high-pressure and low-pressure peak detectors.

The monitoring system estimates three metrics: PIP, PEEP (or the minimum pressure of the breath cycle for assisted breathing), and RR. All three of these metrics are tracked by detecting inhalation and exhalation cycles from the pressure envelopes, as shown in Fig. 6.

The two envelope trackers each store the most recent pressure sample that triggered their attack mode, as shown in the figure. During each inhalation cycle, there are several attack-mode samples in a row for the high-pressure envelope. During exhalation, there are several attack-mode samples in a row for the low-pressure envelope. The system tracks breath cycles by looking for low-pressure attack events that follow high-pressure attack events and vice versa. A low-pressure attack event causes the system to switch from inhalation to exhalation mode, and a high-pressure attack event causes it to switch from exhalation to inhalation mode.

### A. PIP and PEEP

When a mode switch occurs, the previous attack value is used to update the corresponding PIP or PEEP estimate. That is, when a low-pressure attack event occurs, the PIP display is updated with the most recent high-pressure attack value. When a high-pressure attack event occurs, the PEEP display is updated with the most recent low-pressure attack value. Let $V_{\mathrm{high}}[n]$ and $V_{\mathrm{low}}[n]$ be the peak values of the high- and low-pressure envelopes, respectively, during breath cycle $n$, and let $T_{\mathrm{high}}[n]$ and $T_{\mathrm{low}}[n]$ be the sample indices at which they occur.

Both PIP and PEEP are recursively smoothed over time to remove small fluctuations:

\begin{align*}
\mathrm{PIP}[n] & =\alpha _{S}\mathrm{PIP}[n-1]+(1-\alpha _{S})V_{\mathrm{high}}[n]\tag{3}
\\
\mathrm{PEEP}[n] & =\alpha _{S}\mathrm{PEEP}[n-1]+(1-\alpha _{S})V_{\mathrm{low}}[n], \tag{4}
\end{align*}

where $\alpha _{S}$ is a smoothing coefficient between 0 and 1. This is a linear filter with an exponential impulse response; the contribution of sample $n_0$ to the moving average decays as $\alpha _S^{(n-n_0)}$. The closer $\alpha _S$ is to 0, the more quickly the display will respond to changes. We used $\alpha _{S}=0.5$ in our implementation.

### B. Respiratory Rate

The system also keeps track of the time elapsed between these mode-switch events. A complete breath cycle is measured between high-pressure peaks. After smoothing, the average number of samples per breath is

\begin{equation*}
T_b[n] = \alpha _S T_b[n\!-\!1] + (1-\alpha _S)(T_{\mathrm{high}}[n]-T_{\mathrm{high}}[n\!-\!1]). \tag{5}
\end{equation*}

Then the respiratory rate in breaths per minute is given by

\begin{equation*}
\mathrm{RR}[n]=\frac{60 f_s}{T_b[n]}, \tag{6}
\end{equation*}

where $f_{s}$ is the pressure sensor sample rate in samples per second. Note that although they are described mathematically as signals, in practice $T_{\mathrm{high}}$ and $T_{\mathrm{low}}$ are implemented as counters that reset on each breath cycle, as described in Section VII.

**Fig. 7.**
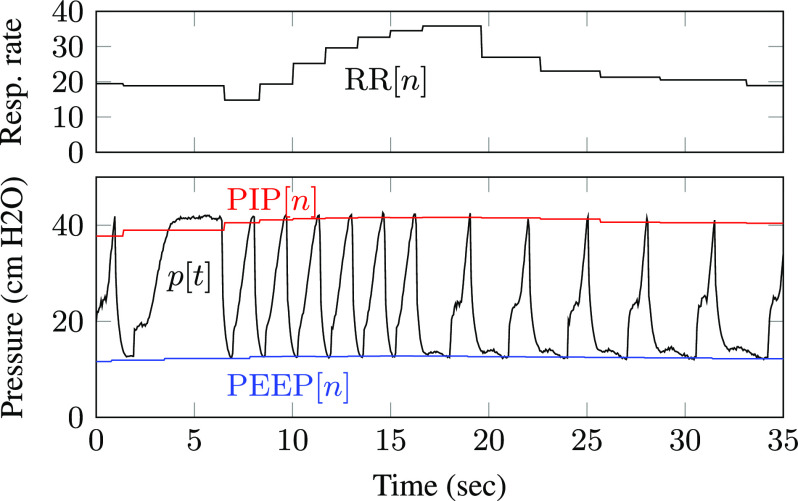
Displayed PIP, PEEP, and respiratory rate values for experimental data from an artificial lung.

Figure 7 shows estimated PIP, PEEP, and respiratory rate values superimposed on a pressure waveform measured using the Illinois RapidVent connected to an artificial lung. Unlike the pressure envelopes, the displayed measurements are updated only once each breath cycle. Smoothing over multiple breaths prevents the values from changing too quickly, as observed in the respiratory rate plot.

## VI. Alarm Conditions

The monitoring device triggers alarms in several conditions that indicate the ventilator is not working properly, as shown in Table I. The alarm thresholds may vary between patients and between ventilator devices and so they are configurable by the user. The table shows the range of values that users of the Illinois RapidVent may select; these were chosen in consultation with local intensive-care experts.

### A. Pressure and Respiratory Rate

The high- and low-pressure alarms trigger immediately if the sensor detects a pressure outside the permitted range. In a pressure-cycled ventilator, the pressure should never exceed the PIP value set by the user. A pressure reading above the range of the PIP dial indicates a mechanical failure. The low-pressure threshold $p_{\mathrm{min}}$ can be set close to zero, that is, atmospheric pressure, to detect a disconnect in the breathing circuit. Note that because pressure-cycled ventilators apply positive pressure even during exhalation, the airway should never drop to atmospheric pressure unless the patient is attempting to breathe spontaneously.

The high- and low-respiratory-rate alarms trigger if the average respiratory rate falls outside the range specified by the user. A high respiratory rate could indicate a low tidal volume, for example due to deteriorating lung compliance, that requires a clinician's attention. The low-respiratory-rate alarm has some overlap with the noncycling alarm, but it triggers based on the average time between complete breath cycles, while the noncycling alarm is triggered by the time elapsed since the last breath event.

### B. Noncycling Conditions

**Fig. 8.**
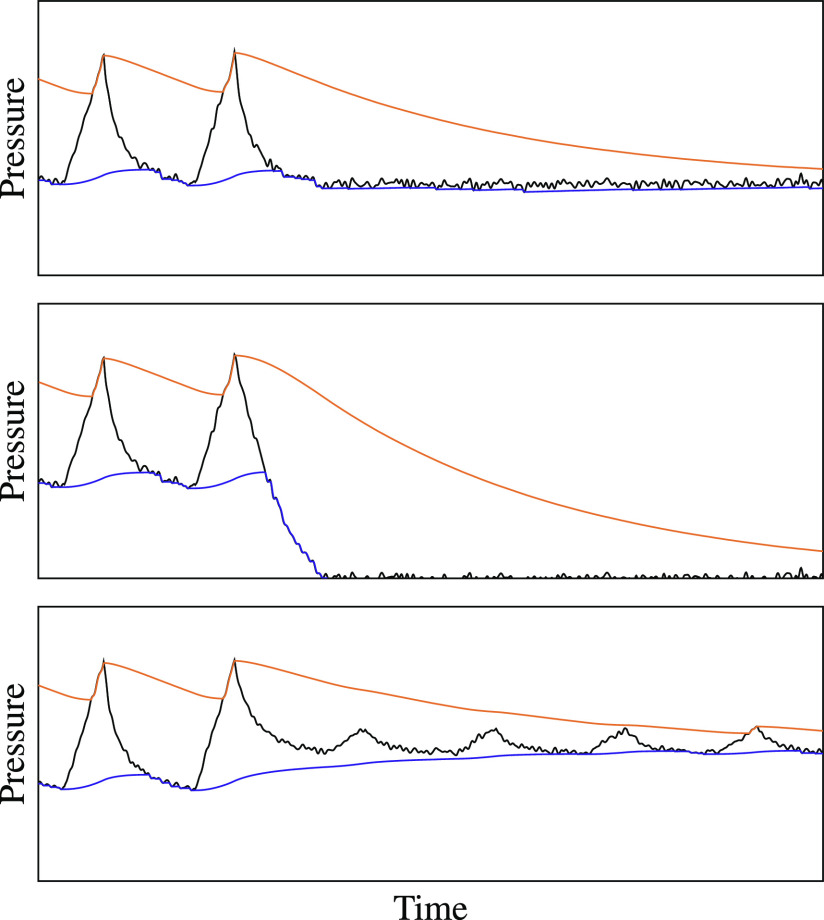
Different pressure signals that should trigger a noncycling alarm.

The noncycling alarm condition is more complex than the first four. It must detect when the breathing cycle has stopped, which can happen in several ways, as illustrated in Fig. 8. Thus, the alarm can be triggered by several conditions.

First, the alarm triggers if too much time has passed since the last attack event of either envelope. For example, if the pressure drops to PEEP and remains constant, as shown in the top panel of Fig. 8, there will be no attack events in the high-pressure envelope tracker, so it will trigger the alarm. If, however, the pressure fluctuates slightly over time, as shown in the bottom panel, the tracking algorithm will still detect frequent peaks.

To handle this case, the alarm will also trigger if the high-pressure envelope and low-pressure envelope are too close together. In pressure-cycled ventilators, the ratio between PIP and PEEP is a constant, here denoted $r_{\mathrm{nom}}$, determined by the mechanical design of the device. For the Illinois RapidVent, the nominal ratio is around 2.4. An alarm is triggered if $v_{\mathrm{high}}[t]/v_{\mathrm{low}}[t]$ drops below $r_{\mathrm{min}}$, a pressure-ratio threshold between 1 and $r_{\mathrm{nom}}$. The alarm also triggers if the difference $v_{\mathrm{high}}[t] - v_{\mathrm{low}}[t]$ is too small. In our implementation, this minimum difference is fixed at 3 cm H$_{2}$O.

### C. Parameter Selection

Because the pressure envelopes $v_{\mathrm{high}}[t]$ and $v_{\mathrm{low}}[t]$ naturally move toward each other during most of the breath cycle, the release coefficient $\alpha _{R}$ and the high-to-low-pressure-ratio threshold $r_{\mathrm{min}}$ jointly determine when the high-to-low-pressure-ratio alarm will trigger. This alarm condition can detect several types of ventilator malfunctions with low computational complexity. However, it is also prone to false positives and false negatives and its parameters must be carefully calibrated.

**Fig. 9.**
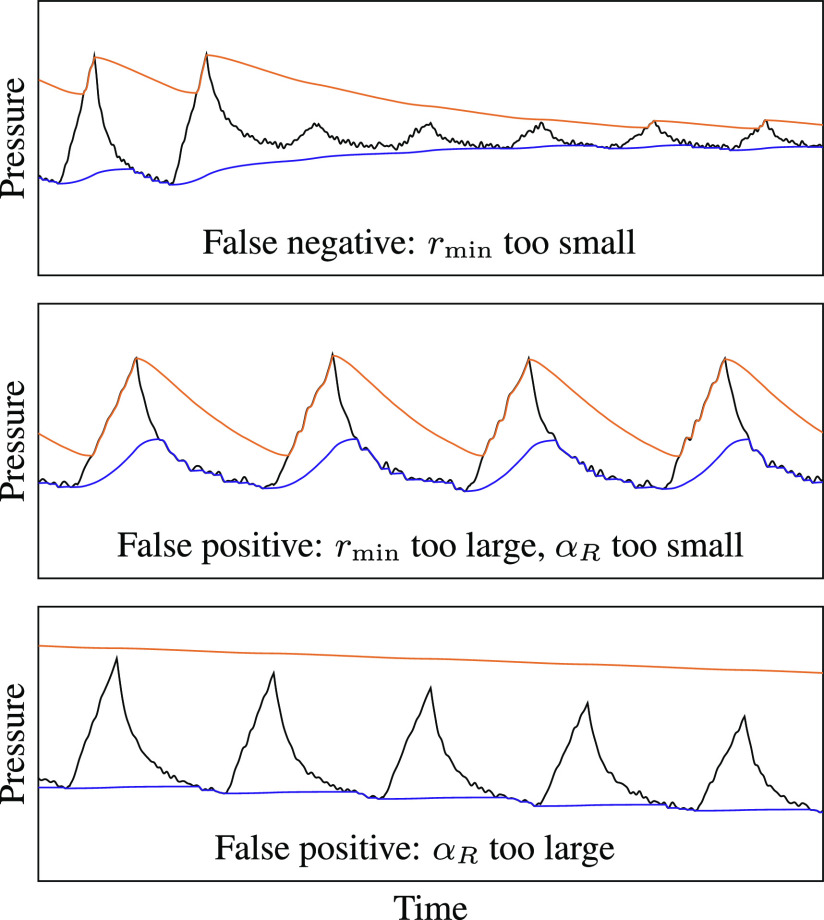
The parameters $\alpha _{R}$ and $r_{\mathrm{min}}$ must be carefully chosen to avoid several types of detection errors.

Figure 9 shows three types of errors that could result from poorly chosen values of $\alpha _{R}$ and $r_{\mathrm{min}}$. First, if $r_{\mathrm{min}}$ is too small—that is, if it is close to 1—the system could mistake noise in the pressure signal for breath cycles. The alarm would then fail to trigger in the event of an obstruction that prevents the ventilator from cycling but still maintains a positive pressure. However, if $r_{\mathrm{min}}$ is too large—that is, close to $r_{\mathrm{nom}}$—it could trigger false alarms during normal breath cycles as the two envelopes fluctuate, as shown in the middle of the figure.

The false-positive ratio alarm could be prevented by increasing the value of $\alpha _{R}$ to make it closer to 1. A larger release coefficient causes the envelopes to decay more slowly between breaths, so that the ratio $v_{\mathrm{high}}[t]/v_{\mathrm{low}}[t]$ varies less over the breath cycle. However, large values of $\alpha _{R}$ can impair the ability of the system to adapt to changes in the pressure signal. For example, if a clinician lowers the PIP setting using the dial on the ventilator, as shown in the bottom panel of Fig. 9, the high-pressure envelope could miss the peaks of several breath cycles. These missed attack-mode samples could falsely trigger the time-since-last-peak alarm and would also cause errors in the PIP, PEEP, and RR calculations.

These three cases illustrate the tradeoffs involved in the selection of $\alpha _{R}$ and $r_{\mathrm{min}}$. To prevent the second error type, the false alarm within a normal breath cycle, we can constrain the relationship between the two parameters: as $r_{\mathrm{min}}$ increases, $\alpha _{R}$ must also increase. In our implementation, we have set $\alpha _{R}$ so that, if the pressure were to fall suddenly from PIP to PEEP and remain constant at PEEP, the noncycling alarm's high-to-low-envelope-ratio condition ($v_{\mathrm{high}}[t]/v_{\mathrm{low}}[t]< r_{\mathrm{min}}$) would be triggered at around the same time as its time-since-last-peak condition ($t-T_{\mathrm{high}}[n]>T_{\mathrm{max}}$) for the default alarm setting of $T_{\mathrm{max}}=15$ seconds. In this scenario, the high-pressure envelope decays as

\begin{equation*}
v_{\mathrm{high}}[t]=\mathrm{PEEP}+\alpha _{R}^{t-T_{\mathrm{high}}}(\mathrm{PIP}-\mathrm{PEEP}). \tag{7}
\end{equation*}

The alarm will therefore be triggered when

\begin{equation*}
v_{\mathrm{high}}[t]=r_{\mathrm{min}}\times \mathrm{PEEP}, \tag{8}
\end{equation*}

or

\begin{equation*}
r_{\mathrm{min}}=1+\alpha _{R}^{t-T_{\mathrm{high}}}\left(\frac{\mathrm{PIP}}{\mathrm{PEEP}}-1\right). \tag{9}
\end{equation*}

Setting the elapsed time to the alarm time $T_{\mathrm{max}}$ and solving for $\alpha _{R}$, we have

\begin{equation*}
\alpha _{R}=\left(\frac{r_{\mathrm{min}}-1}{r_{\mathrm{nom}}-1}\right)^{1/T_{\mathrm{max}}}. \tag{10}
\end{equation*}

Having fixed $\alpha _{R}$ as a function of $r_{\mathrm{min}}$, we must choose $r_{\mathrm{min}}$ to trade off between false positives and false negatives. Choosing small values of $r_{\mathrm{min}}$ and $\alpha _{R}$ can lead to false negatives, as shown in the top panel of Fig. 9. Choosing large values can lead to false positives, as shown in the bottom panel. Because false negatives are worse than false positives in a life-support device, $r_{\mathrm{min}}$ should be chosen to be comfortably larger than 1. In our implementation, we use $r_{\mathrm{min}}=1.5$.

**Fig. 10.**
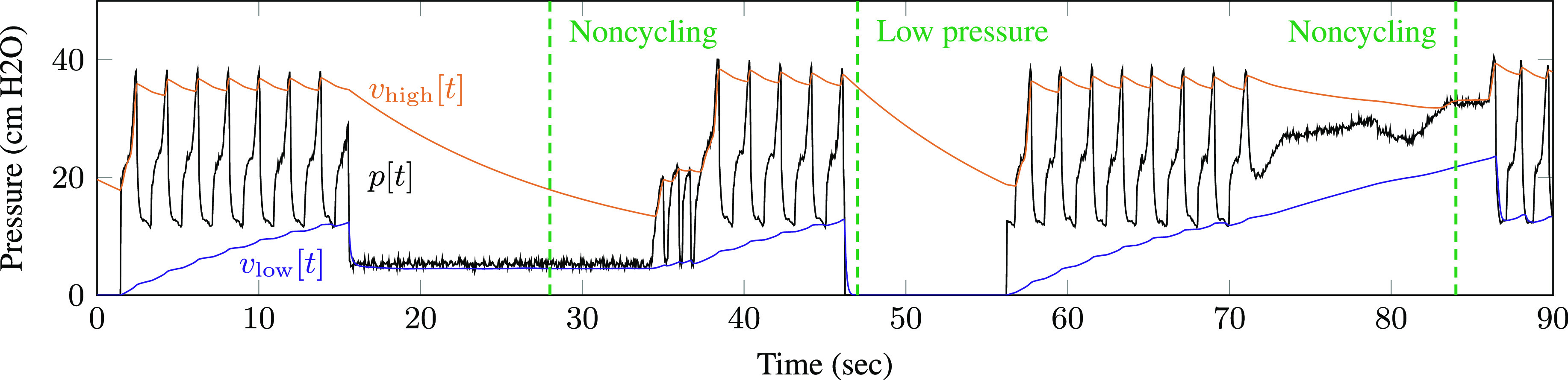
Alarm-triggering conditions were simulated with an artificial lung. The dashed lines indicate alarm events.

Figure 10 shows alarms triggered by experimental data from the Illinois RapidVent connected to an artificial lung. The ventilator was obstructed and disconnected to create noncycling conditions. Notice that the low-pressure alarm triggers immediately when the breathing circuit is disconnected, while the noncycling alarm triggers after 15 seconds based on the user-specified alarm threshold.

## VII. Algorithm Implementation

Algorithm 1: **Ventilator Monitoring Algorithm**
**loop**
   Read $p$ from pressure sensor
   $T_{\mathrm{peak}} \gets T_{\mathrm{peak}}+1$
   
   **if** $p \geq v_{\mathrm{high}}$ **then**
      $v_{\mathrm{high}} \gets \alpha _A v_{\mathrm{high}} + (1-\alpha _A) p$
      $V_{\mathrm{high}} \gets p$
      $T_{\mathrm{high}} \gets 0$
      **if** breath state $=$ exhaling **then**
         breath state $\gets$ inhaling
         $\mathrm{PEEP} \gets \alpha _S \mathrm{PEEP} + (1-\alpha _S) V_{\mathrm{low}}$
      **end if**
   **else**
      $v_{\mathrm{high}} \gets \alpha _R v_{\mathrm{high}} + (1-\alpha _R) p$
      $T_{\mathrm{high}} \gets T_{\mathrm{high}} + 1$
   **end if**
   
   **if** $p \leq v_{\mathrm{low}}$ **then**
      $v_{\mathrm{low}} \gets \alpha _A v_{\mathrm{low}} + (1-\alpha _A) p$
      $V_{\mathrm{low}} \gets p$
      $T_{\mathrm{low}} \gets 0$
      **if** breath state $=$ inhaling **then**
         breath state $\gets$ exhaling
         $\mathrm{PIP} \gets \alpha _S \mathrm{PIP} + (1-\alpha _S) V_{\mathrm{high}}$
         $\mathrm{RR} \gets \left(\alpha _S \mathrm{RR}^{-1} + (1-\alpha _S)\frac{T_{\mathrm{peak}} - T_{\mathrm{high}}}{60f_s}\right)^{-1}$
         $T_{\mathrm{peak}} \gets T_{\mathrm{high}}$
      **end if**
   **else**
      $v_{\mathrm{low}} \gets \alpha _R v_{\mathrm{low}} + (1-\alpha _R) p$
      $T_{\mathrm{low}} \gets T_{\mathrm{low}} + 1$
   **end if**
   
   Check alarm conditions from Table I
**end loop**

• $p$ – current pressure sample
• $v_{\mathrm{high}}$ – high-pressure envelope
• $v_{\mathrm{low}}$ – low-pressure envelope
• $V_{\mathrm{high}}$ – breath-cycle maximum
• $V_{\mathrm{low}}$ – breath-cycle minimum
• $T_{\mathrm{high}}$ – samples since most recent breath-cycle maximum
• $T_{\mathrm{low}}$ – samples since most recent breath-cycle minimum
• $T_{\mathrm{peak}}$ – samples since previous breath-cycle maximum
• $\mathrm{PIP}$, $\mathrm{PEEP}$, $\mathrm{RR}$ – smoothed PIP, PEEP, and RR output
• $\alpha _A$, $\alpha _R$ – envelope attack and release coefficients
• $\alpha _S$ – smoothing coefficient
• $f_s$ – sample rate (samples per second)

The monitoring algorithm can be implemented with low computational complexity and a small memory footprint. The core loop of the algorithm is shown in Algorithm 1. The algorithm state comprises a binary inhalation/exhalation state variable; seven floating-point values, the envelopes $v_{\mathrm{high}}$ and $v_{\mathrm{low}}$, the breath-cycle peaks $V_{\mathrm{high}}$ and $V_{\mathrm{low}}$, and the estimated PIP, PEEP, and RR; and the three integer counters $T_{\mathrm{peak}}$, $T_{\mathrm{high}}$, and $T_{\mathrm{low}}$. The program must also store the user-configurable alarm thresholds in memory.

For every observed sample, the envelopes are updated according to (1) and (2) and the alarm conditions from Table I are checked. Breaths are tracked using a state variable that toggles from inhalation to exhalation at the first low-pressure attack value in each breath cycle and from exhalation to inhalation at the first high-pressure attack value in each cycle. The time since the last attack for each envelope is tracked using counters $T_{\mathrm{high}}$ and $T_{\mathrm{low}}$. Thus, the time-based noncycling alarm conditions from Table I can be written $T_{\mathrm{high}}>T_{\mathrm{max}}$ and $T_{\mathrm{low}}>T_{\mathrm{max}}$. An additional counter $T_{\mathrm{peak}}$ counts the samples since the previous high-pressure peak (the circles in Fig. 6), which allows the system to compute the time between breath cycles. The PIP, PEEP, and RR metrics are each updated once per breath cycle, regardless of the sample rate.

To assess the computational complexity of the system, the execution time of the algorithm was measured on the ATmega328 with a clock speed of 8 MHz. Table II shows storage, memory, and execution time of the monitoring algorithm and of the user interface logic that controls the buttons, buzzer, and display. The monitoring algorithm requires less than one millisecond per sample. The memory and storage requirements are dominated by the user interface logic.

**TABLE II:** Resource Utilization

	Program storage (bytes)	Dynamic memory (bytes)	Execution time per sample ($\mu$s)
Interface	6847	357	146
Algorithm	4048	93	670
Total	10895	450	816

Because the recursive envelope tracker performs a fixed number of calculations for each pressure sample, the overall computational complexity of the system depends on the sample rate. To select an appropriate sample rate for the monitoring system, we must characterize the performance of the algorithm at different sample rates. If the sample rate is too low, the sampled sequence might not capture the narrow peak of the pressure waveform, causing errors in the estimated PIP and RR values.

Table III shows the root-mean-square error in PIP and RR measurements when the algorithm is run at different sample rates. The pressure data is from an artificial lung cycling at a rate of more than 30 breaths per minute, which is faster than typical human respiratory rates. The error at lower sample rates is calculated relative to estimates performed at a baseline rate of 100 samples per second. Even for these fast breaths, the estimated PIP and RR values are accurate within the display resolution of 1 cm H$_{2}$O and 1 breath/min for sample rates as low as 10 samples per second.

**TABLE III:** Sample Rate and Performance

Sample rate (samples/sec)	RMS PIP error (cm H$_{2}$O)	RMS RR error (breaths/min)
5	1.7	5.4
10	0.5	0.4
20	0.6	0.2
50	0.3	0.1
100	Baseline	

These results suggest that the sensor and alarm module should use a sample rate of at least 10 samples per second. This rate is well within the capability of most modern microcontrollers, even low-cost 8-bit processors that must use many clock cycles to perform floating-point calculations.

## VIII. Animal Testing

The monitoring algorithm was validated using animal-testing data collected during the development of the Illinois RapidVent. The prototype ventilator was tested using sedated pigs, which have lungs that are similar in size to those of humans [19], [20]. The tests were conducted at the University of Illinois at Urbana-Champaign under protocol number 20071 approved by the Institutional Animal Care and Use Committee. Details are given in an upcoming paper about the Illinois RapidVent [10]. The primary purpose of the tests was to evaluate the performance of the ventilator itself and the experiment did not include the prototype monitoring device. However, the pigs’ airway pressure was measured continuously during the tests using a Rosemount 3051S differential pressure transducer sampled about seven times per second. The experiments included multiple combinations of PIP and rate dial settings, both mandatory and assisted breathing, and accidental and deliberate disconnections. Therefore, the pressure data captured during the animal tests are valuable for validating the proposed monitoring algorithm.

**Fig. 11.**
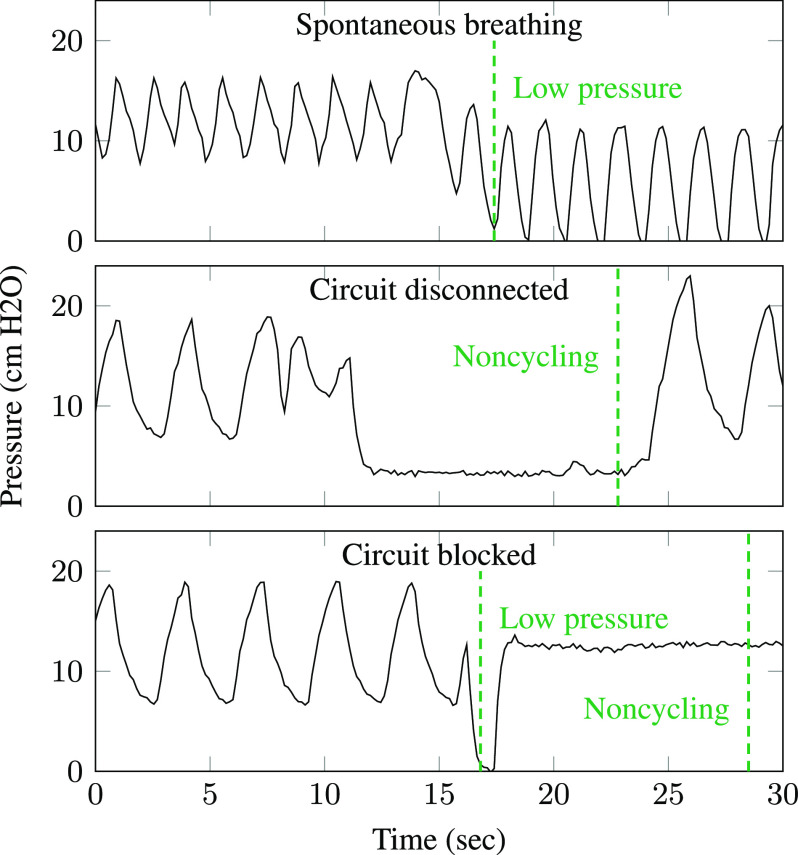
The monitoring algorithm was validated using data from a pressure-cycled ventilator on a sedated pig. Dashed lines indicate alarms triggered by the algorithm.

Figure 11 shows excerpts from the pressure data and the alarms triggered by the monitoring algorithm. In the top panel, the pig inhaled strongly enough to pull pressure near zero (atmospheric level), triggering the low-pressure alarm. This alarm does not indicate ventilator failure, but alerts clinicians that the patient is breathing spontaneously. The middle panel shows a disconnection event: the pig rolled over, breaking the respiratory circuit. The pressure did not drop to atmospheric level but remained at a steady low level, triggering the noncycling alarm. In the bottom panel, the ventilator was deliberately blocked for several seconds in order to measure tidal volume, leading to a momentary drop and then steady high pressure level. This event triggers both the low-pressure and noncycling alarms. The envelope-tracking algorithm was found to work for all tested settings of the PIP and rate dials, although sudden changes in the dial settings can trigger false alarms and cause temporary inaccuracies in metric estimates.

## IX. Conclusion

The proposed sensor and alarm system can improve the functionality of pressure-cycled emergency ventilators. While it is not as robust as a full-featured commercial ventilator system, it provides critical monitoring features that are not available on purely mechanical ventilators. The recursive envelope-tracking algorithm allows the system to track breathing, estimate metrics, and detect malfunctions with only a few calculations per sample and a tiny memory footprint. Therefore, the system can be built quickly using nearly any low-cost microcontroller and a few other electronic components.
